# What factors influence the length of stay and readmission after deep brain stimulation surgery? a tertiary centre study

**DOI:** 10.1007/s00701-026-06855-x

**Published:** 2026-04-15

**Authors:** Jason Yuen, Emily Boyd, Madelaine Miller, Adam Williams, Reiko Ashida

**Affiliations:** 1 Department of Neurosurgery, Southmead Hospital, Bristol, BS10 5NB UK; 2 University of Bristol, Bristol, BS8 1 UK

**Keywords:** Deep brain stimulation, Length of stay, Readmission, Parkinson’s disease, Essential tremor, Dystonia

## Abstract

**Purpose:**

Deep Brain Stimulation (DBS) is a well-established treatment for refractory movement disorders. However, there are surgical risks and it often includes in-patient hospital admission. The aim is to review key factors associated with prolonged length of stay (LoS)(defined here as > 2 nights), and readmission within six months.

**Methods:**

We retrospectively reviewed medical records of patients who underwent DBS insertion between October 2016 and September 2024 in our tertiary centre. Patient and operative factors were reviewed.

**Results:**

397 DBS procedures(388 patients) were included. Parkinson’s disease (PD) patients constitute majority(73%), followed by Essential Tremor(13%). Mean LoS were 2.39 ± 0.2 and 2.48 ± 0.5 nights, respectively. Within PD cohort, older age and use of blood thinners were associated with increased LoS. Other factors such as gender, baseline Unified Parkinson's Disease Rating Scale-3 score, symptom duration, and operating time were not statistically significant. Readmission rate was 10.3%(41/397), with majority secondary to infection(20/41) or planned readmission(13/41). Excluding planned readmissions, average LoS during readmission was 13.4 ± 0.6 nights, with majority readmitted > 30 days post-discharge (15/27). Within PD, readmission rate was 6.9%(19/276), with skull-mounted implant use identified as risk factor.

**Conclusions:**

This study identified risk factors for prolonged hospital stay after DBS surgeries, with our surgical workflow, in a publicly-funded healthcare system. We also captured factors associated with readmission within six months, which is a much longer timeframe than most studies in literature. This provides information to facilitate prehabilitation, resource allocation, and patient counselling to optimise patient outcome and reduce treatment costs. Further studies are warranted to confirm these findings, especially with different DBS techniques and workflows.

## Introduction

**Fig. 1:**
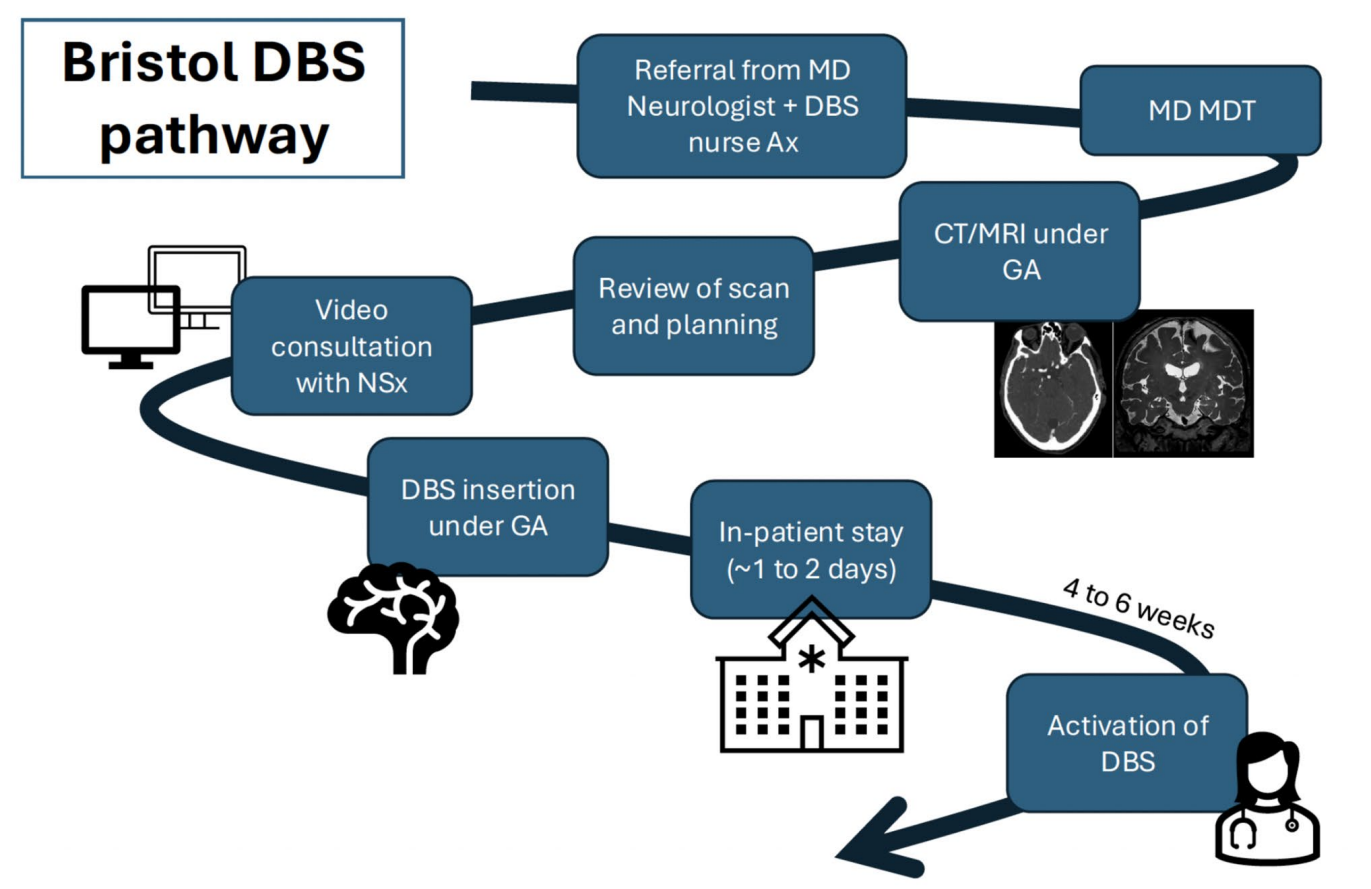
DBS workflow at our tertiary centre. Icons from Microsoft Stock Images. Ax, assessment; CT, computed tomography; DBS, Deep Brain Stimulation, GA, general anaesthesia; MD, movement disorder; MDT, Multidisciplinary team meeting; MRI, magnetic resonance imaging; NSx, Neurosurgery

Deep brainstimulation (DBS) is a well-recognised procedure for a number of movement disorders, such as Parkinson’s disease (PD) and essential tremor (ET), as well as experimental indications in neuropsychiatric diseases [24, 26, 27, 29, 34, 42, 52, 53]. While the key objective essentially involves the insertion of electrodes into deep parts of the brain for neuromodulatory purposes, there are a number of different methodologies for achieving that, such as use of awake or asleep surgery, as well as different hardware [43]. The workflow also varies from unit to unit. The workflow of our unit is given in Fig. 1. From our experience, patients typically have an in-patient stay for one to two nights post-operatively but some may have to stay longer due to a range of reasons (which we will explore further in this study).

It is important to identify risk factors associated with prolonged in-patient stay for two important reasons. First, resources are limited in healthcare systems, particularly in publicly-funded system, such as the National Health Service (NHS) in the United Kingdom. An average DBS procedure cost (including device) is approximately USD$30 000-$80 000 [4, 20]. Extra hospital stay would incur additional costs. The cost of a standard bed stay in the NHS is approximately £350 [15, 38]. Further, the cost of additional therapy such as physiotherapy, occupational therapy, imaging, and neurology consult would incur significant morbidity and costs. The second reason is the downstream effect of prolonged stay leading to cancellation of elective procedures, due to lack of in-patient bed availability [41]. Identifying risk factors associated with prolonged length of stay (LoS) and those associated with readmission may potentially offer better resource allocation, patient counselling, and opportunities to prehabilitate these patients by optimising their medical status. The aim of the current study is to identify potential risk factors associated with prolonged length of stay (which we define as more than two nights) and readmission within six months after DBS surgery.

## Methods

Data was collected retrospectively using hospital medical records over eight years (October 2016-September 2024). Replacements of implantable pulse generator (IPG) alone were excluded. Study was registered with North Bristol NHS Trust Clinical Audit and Effectiveness Department. Data collection included.

- demographics (age, sex),
- patient-specific factors (use of antiplatelets/anticoagulation, immunosuppression, pre-operative Parkinson's Disease Questionnaire-39 (PDQ-39) mobility score, pre-operative Dementia Rating Scale-2 (DRS-2) scores reported as Age and Education Corrected Moan’s Scaled Score (AEMSS), multiple deprivation index, distance from home to hospital using postcode),
- disease-specific factors (disease duration, disease-specific scores),
- operation-specific factors (American Society of Anesthesiologists (ASA) grade, operation duration), and
- length of stay during surgery.

Disease-specific scores included Unified Parkinson's Disease Rating Scale-3 (UPDRS3) score for PD patients and Clinical Tremor Score (CTS) for ET patients. For patients who stayed for more than 2 nights, reasons for prolonged stay were also recorded. Also, for patients who were readmitted within six months of discharge, reasons for readmission were explored.

Regarding surgical technique, our centre uses a robot-assisted delivery of electrodes under general anaesthesia without intra-operative microelectrode recording. Further details of the surgical technique can also be found in a previous publication [32]. In our institute. targeting mostly involves subthalamic nucleus (STN) for PD cases. If it is tremor dominant PD, we also try to capture part of the caudal zona incerta (cZI) in target selection by positioning the electrode in a more posterior, medial and deeper position, relative to the STN. For ET cases, we would usually target exclusively the cZI. In terms of hardware, we predominantly use the standard DBS implants from the major companies (Boston Scientific, Medtronic, Abbott), as well as a small proportion with the skull-mounted device (Picostim) as part of a clinical trial [7].

Statistical analysis was performed using IBM SPSS Statistics for Mac, Version 29.0 (Armonk, NY: IBM Corp). Univariate analyses were performed using Student *t-*test (age, duration of symptoms, operating time, distance to hospital), χ^2^ test (gender in PD, use of antiplatelets/anticoagulation, immunosuppression, smoking), Fisher test (gender in ET), and Mann–Whitney test (length of stay with readmission, UPDRS3, CTS, ASA grade, PDQ39 mobility score, adjusted Automated Memory and Executive Screening (AMES) score, multiple deprivation index). In summary, Pearson’s χ^2^ test was used for comparison of categorical values; Mann–Whitney U test was used to compare ordinal and continuous values between groups (since LoS does not follow a normal distribution). Two-sided tests were used. Multivariate analyses were performed using binary logistic regression model with the same predicting variables. Linear regression modelling was used to explore correlation between continuous variables and LoS. Results where *p*-value < 0.05 were considered statistically significant.

## Results

**Fig. 2:**
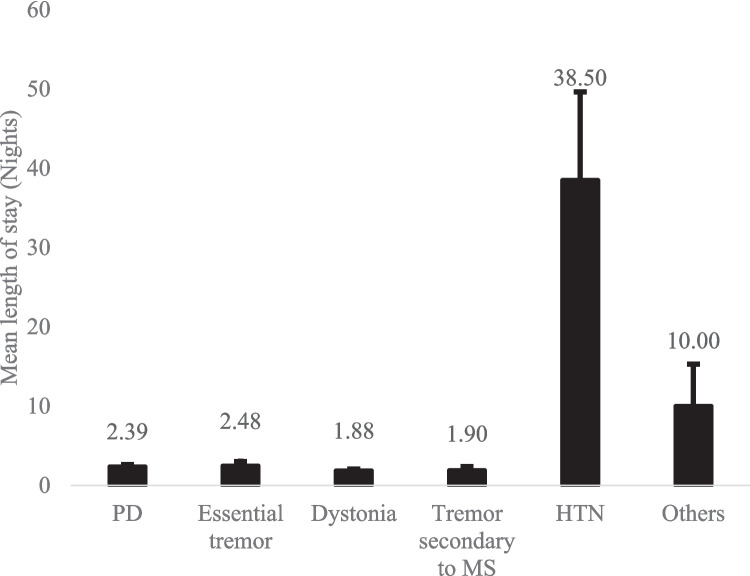
Mean length of stay for patients who underwent Deep Brain Stimulation surgery. HTN, hypertension; MS, multiple sclerosis, PD, Parkinson’s disease. The “Other” category includes one patient from each of: pain, obsessive–compulsive disorder, and Anti-MAG demyelinating neuropathy. Error bars denote standard error

Data was collected for 397 operations (388 patients). 34% were female. The two largest groups were PD (73%) and ET (13%) patients. The mean LoS for different indications is given in Fig. 2. Of note, DBS for hypertension is an ongoing clinical trial run in the Department. Patients in this category had a longer LoS due to need for medication adjustments and intense early programming need.

Considering only the two largest groups of patients (PD and ET), a proportion of patients had multiple reasons documented for prolonged post-operative stay. The most commonly cited reasons were mobility issues (*N* = 25), such as difficulty mobilising, unsteadiness, falls, and hypotension. The next commonest reasons were (*N* = 13 each) autonomic dysfunction, *i.e.* constipation and urinary retention, and patients coming from abroad. Historically, patients from the Republic of Ireland visited our unit for DBS surgery but this arrangement has now ceased. These patients tended to have prolonged admission time due to logistic rather than clinical reasons.

Statistical analysis of individual variables in the PD and ET cohort was performed (Tables 1 and 2), excluding patients from Republic of Ireland. Among PD patients, mean LoS was 2.39 ± 0.2 nights. Among short stayers (SL), mean LoS was 1.48 nights [median 1, range 1–2]. Long stayers (LL) had a mean LoS of 6.16 nights [median 3, range 3–50]. Older age and use of antiplatelets/anticoagulation were both associated with prolonged LoS, with statistical significance. Linear regression models correlating LoS with Age, UPDRS3, Operating time, AEMSS, PDQ39, Distance to hospital yielded an R^2^ of 0.062, with only Age having a positive association with LoS (*p* < 0.021).

**Table 1:** Statistical analysis of factors affecting length of stay of patients with Parkinson’s disease (PD) undergoing Deep Brain Stimulation (DBS)

PD length of stay	Results		*p*-value (univariate)	*p*-value (multivariate)
	SL (225)	LL (51)		
Age (yrs)	61.2 ± 0.5	64.3 ± 1.3	***0.016***	***0.036***
Gender (M:F)	154:71	36:15	0.765	0.853
UPDRS3	38.7 ± 0.8	40.6 ± 5.7	0.417	0.977
Duration of symptoms (yrs)	10.3 ± 0.3	11.4 ± 0.7	0.110	0.137
ASA grade (median and range)	3 [1–4]	3 [2–3]	0.056	0.420
Antiplatelets/anticoagulation	211 on nothing	38 on nothing	** < *0.001***	** < *0.001***
Smoking	111 non-smoker	26 non-smoker	0.472	0.535
PDQ39 mobility	18.5 ± 0.7	20.6 ± 2.9	0.183	0.200
AEMSS	12.4 ± 0.2	11.7 ± 1.7	0.055	0.418
Immunosuppression (suppressed)	16	5	0.513	0.366
Multiple deprivation index	6.5 ± 0.2	6.2 ± 0.9	0.430	0.362
Distance to hospital (km)	85.2 ± 4.6	98.3 ± 14.3	0.251	0.112
Involvement of cZi	123/225 (54.7%)	31/51 (60.8%)	0.440	0.520
Proportion of conventional vs skull-mounted device	14/225 (6.2%)	3/51 (5.9%)	1.000	0.617
Operating time	2:44 ± 0:03	2:51 ± 0:24	0.399	0.725

*AEMSS* Age and Education Corrected Moan’s Scaled Score; cZi caudal zona incerta, *LL* Longer length of stay (> 2 nights), *NA* Not available, *PDQ-39* Parkinson's Disease Questionnaire-39, *SL* Standard length of stay (≤ 2 nights), *UPDRS* Unified Parkinson's Disease Rating Scale. PD cases exclude 13 patients from abroad. Standard errors (S.E.s) were given where indicated. 88 patients excluded in multivariate analysis due to missing values

**Table 2:** Statistical analysis of factors affecting length of stay of patients with Essential Tremor (ET) undergoing Deep Brain Stimulation (DBS)

ET length of stay	Results		*p*-value (univariate)	*p*-value (multivariate)
	SL (42)	LL (7)		
Age (yrs)	66.1 ± 1.9	72.6 ± 2.7	0.176	0.106
Gender (M:F)	30:12	2:5	***0.041***	0.812
CTS	59.4 ± 2.9	58.0 ± 4.5	0.840	0.985
Duration of symptoms (yrs)	24.9 ± 2.9	16.4 ± 3.7	0.089	***0.008***
ASA grade (median and range)	3 [2–3]	3 [2–3]	0.566	0.131
Antiplatelets/anticoagulation	25 on nothing	7 on nothing	0.227	0.280
Smoking	12 non-smoker	4 non-smoker	0.369	***0.004***
AEMSS	12.2 ± 0.4	12.6 ± 0.8	0.900	1.000
Immunosuppression (suppressed)	15	2	0.713	0.244
Multiple deprivation index	6.2 ± 0.4	5.0 ± 1.8	0.511	0.905
Distance to hospital (km)	93.4 ± 14.1	128.1 ± 57.2	0.399	0.195
Operating time	2:43 ± 0:06	3:10 ± 0:17	0.120	***0.012***

*AEMSS* Age and Education Corrected Moan’s Scaled Score, *LL* Longer length of stay (> 2 nights), *CTS* Clinical tremor score, *NA* Not available, *SL* Standard length of stay (≤ 2 nights). None of the ET patients were from Ireland. Standard errors (S.E.s) were given where indicated. 14 patients excluded in multivariate analysis due to missing values

Among ET patients, mean LoS was 2.48 ± 0.5 nights. SL had a mean LoS of 1.55 nights [median 2, range 1–2]. LL had a mean LoS of 8.29 nights [median 3, range 3–22]. Female patients were associated with a higher risk of prolonged LoS in univariate analysis but this was not positive in subsequent multivariate analysis. Vast majority of cases targeted the Zi and none involved the skull-mounted device. On the other hand, multivariate analysis showed a shorter duration of symptoms, a positive smoking history, and longer operating duration were associated with longer hospital stay. Linear regression models correlating LoS with Age, CTS, Operating time, AEMSS, PDQ39, Distance to hospital yielded an R^2^ of 0.134, and none of the factors having a positive association with LoS.

**Fig. 3:**
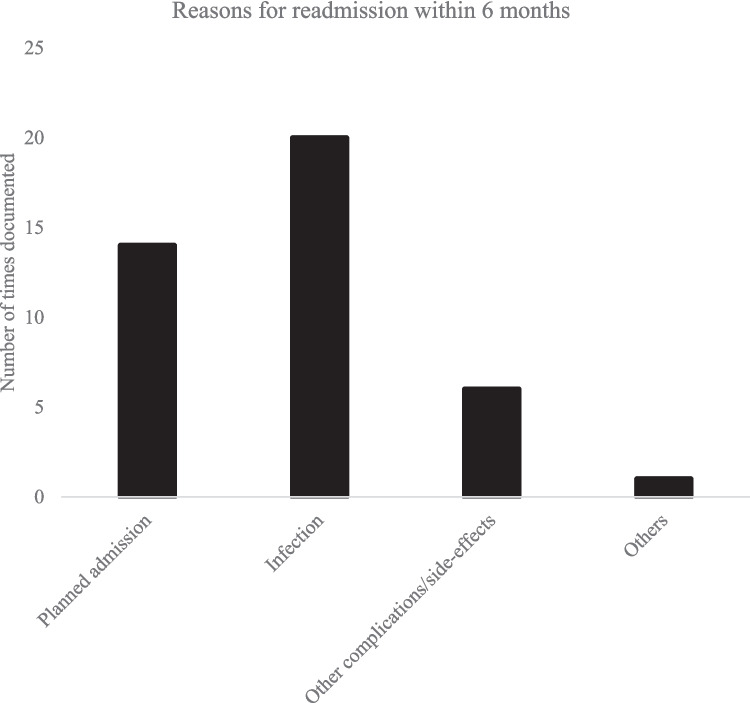
Documented reasons for readmission within 6 months for all indications. Some patients had more than one indication

In terms of overall readmission data, most patients were PD patients (78%), followed by dystonia (7%), and ET (5%). The causes of readmission (*N* = 41) are given in Fig. 3, with some patients having multiple causes. Infection (including ones that were treated prophylactically without microbiological confirmation) is the commonest cause (*N* = 20; 5.0% of all cases) but only 9/20 (2.2% of all cases) required explantation of device. There were 14 patients who were admitted as planned readmissions for programming purpose and these were excluded in subsequent analysis. Mean LoS during *re*admission was 13.4 ± 0.6 days. Mean duration between discharge and readmission was 38.1 ± 5.8 days. 15 out of 27 patients were readmitted more than 30 days post-discharge. Within the PD cohort, readmission rate was 6.9% (19/276). None of the risk factors measured were associated with readmission, except for use of the skull-mounted device (Table 3).

**Table 3:** Statistical analysis of factors affecting readmission within 6 months of patients with Parkinson’s disease (PD) undergoing Deep Brain Stimulation (DBS)

Readmission within 6 months (PD)	Results		*p*-value (univariate)	*p*-value (multivariate)
	*N* (257)	Y (19)		
Age (yrs)	62.0 ± 0.5	59.7 ± 2.2	0.257	0.729
Gender (M:F)	181:76	11:8	0.252	0.289
UPDRS3	39.2 ± 0.8	36.7 ± 1.9	0.463	0.285
Duration of symptoms (yrs)	10.4 ± 0.3	11.2 ± 1.2	0.468	0.567
ASA grade (median and range)	3 [1–4]	2 [2–3]	0.068	0.261
Antiplatelets/anticoagulation	229 on nothing	16 on nothing	0.736	0.226
Smoking	129 non-smoker	8 non-smoker	0.798	0.957
PDQ39 mobility	18.8 ± 0.6	17.9 ± 2.2	0.708	0.455
AEMSS	12.3 ± 0.2	12.2 ± 0.5	0.627	0.347
Immunosuppression (suppressed)	20	1	0.689	0.942
Multiple deprivation index	6.4 ± 0.2	7.0 ± 0.5	0.345	0.198
Involvement of cZi	144/257 (56.0%)	8/19 (42.1%)	0.339	0.923
Proportion of conventional vs skull-mounted device	10/257 (3.9%)	5/19 (26.3%)	***0.002***	** < *0.001***
Operating time	2:44 ± 0:03	2:50 ± 0:16	0.729	0.719
Distance to hospital (km)	85.4 ± 4.8	97.0 ± 11.8	0.320	0.374
Length of stay (days)	2.3 ± 0.2	1.6 ± 0.2	0.156	0.148 (SL vs LL)

*cZi* caudal zona incerta, *NA* Not available, *SL* Standard length of stay (≤ 2 nights), *LL* Longer length of stay (> 2 nights). This excludes 13 planned readmissions for in-patient assessment and programming. 93 patients were excluded in multivariate analysis due to missing values. Standard errors (S.E.s) were given where indicated

## Discussion

The current study provided evidence for risk factors associated with prolonged hospital stay after DBS surgery with our workflow. To our knowledge, no other comparable UK-based studies were published in the literature.

### Prolonged length of stay

In two large US database studies of 6058 and 27,956 patients [23, 45], the mean length of stay was 1.7 to 1.88 days but only 87.6% to 88.8% were discharged home without extra care. In our series, all patients returned to baseline and were discharged home, hence there is some discrepancy in the post-operative care provided. This is likely to be due to the differences in healthcare system (public vs. private). There could potentially be more pressure for patients to be discharged earlier in order to avoid expensive hospital expenses in a private system.

In two smaller studies between 200 to 300 patients in the US [6, 20], the mean LoS was even lower at around 1.19 days. 30-day readmission rate was 1.4% to 4.3%. In one study [6], infection rate was exceptionally low at 1.1% but hardware complication rate was 6.0% in overall, which is higher than expected, given the low infection rate. Infection rate in the literature of large studies ranges between 2.6 to 9.3%, in line with our study [1, 3, 5, 8, 9, 14, 16, 31, 36, 37, 46, 48, 50]. The exceptionally low rate reported by the US study likely represents an outlier.

In the literature, the most commonly cited reasons for delayed discharge included mental status change, haemorrhage, nausea, urinary retention [6, 30]. Also, delayed discharge was linked to worse pre-operative cognitive status (MMSE score), higher pre-surgical “on” motor score and higher number of microelectrode passes [30], as well as indication of obsessive–compulsive disorder, increased physical and psychiatric comorbidity [23]. Nevertheless, pre-operative cognitive status (in the form of DR-2 score) was not found to be a significant factor in our study. This may be due to our strict patient selection process, as well as the use of the more detailed DR-2 score rather than the briefer MMSE score.

Age was a significant factor associated with prolonged stay in PD patients in the current study, which is not surprising given advanced age is associated with increased frailty and slower recovery. In two studies of 1757 and 861 patients respectively, it was found that increasing age (including > 75 years) do not contribute to increased 90-day complication rate or readmission. This is consistent with the paucity of association between readmission and age in the current study [9, 51]. In addition, low-volume centres (< 5 cases per year) were found to be associated with prolonged stay [21]. This does not tend to be an issue in the United Kingdom as most centres that perform DBS would exceed that number.

While it is not possible to alter non-modifiable risk factors such as age, it is useful to explore why use of antiplatelets/anticoagulation is associated with longer hospital stay. In our centre, antiplatelets are typically stopped five to seven days in advance of surgery (anticoagulants are stopped at least 2 days in advance). The underlying reason for this association is not clear. It could be due to underlying confounder such as multiple co-morbidities but it is not reflected in the ASA grading. Further studies will be helpful to confirm that. Nevertheless, our findings may be helpful to manage patient expectations and facilitate counselling in the pre-operative period.

Furthermore, given mobility appears to be the main reason for prolonged in-patient stay, more intensive pre-operative physical therapy may help to optimise the patient’s baseline to minimise the risk, since recovery from general anaesthetics could worsen mobility (especially in the elderly) [44]. Enhanced recovery after surgery (ERAS) protocols may also be beneficial for patients at risk [10]. Another major reason of prolonged stay in the current study is bowel and bladder dysfunction. While those with PD are already at a higher risk of suffering from constipation [2], this can be further exacerbated by post-operative opioids. Interventions such as increased fibre intake and laxatives may reduce the risk. This is especially important since constipation may reduce absorption of PD medications, which may lead to post-operative rigidity and bradykinesia, causing obstruction to early mobilisation [25]. While some centres advocate the use of nasogastric (NG) tubes to administer PD medications during the DBS operation [33], this is not routinely performed in our centre, as it is felt the risks outweigh the benefit. However, a subgroup of patients with “brittle response” PD, where there is a narrow therapeutic window of levodopa response, the use of NG tube may be justified to help to improve post-operative mobility and facilitate prompt discharge. In the literature, brittle response PD is found to be associated with female patients with a low body weight, and those with a longer disease duration and longer duration of levodopa therapy [28]. In addition, our centre also avoids the use of urinary catheter during DBS surgery to minimise risk of post-operative urinary retention but this may be helpful in those who are at risk, e.g., those with history of urinary tract outflow obstruction.

From the literature, targeting the cZI (or known as posterior subthalamic area, PSA) could increase the risk of adverse effects such as ataxia [22]. However, we do not find this to be a risk factor for extended LoS and readmission, supporting its use in PD patients.

Our ET results demonstrated shorter duration of symptoms, positive smoking history, and longer operating time as risk factors for longer hospital stay. However, these were not replicated in the PD cohort. While smoking and longer operating time are traditional surgical risk factors, given the limited number in the ET group, it is important to perform further studies to confirm our results. However, one consideration for prehabilitation may be to focus on encouraging smoking cessation and avoiding prolonged operating time in this cohort. The cohort of dystonia patients was not analysed due to the relative low patient number and heterogenous aetiologies, e.g., primary vs. secondary causes, generalised vs. focal distributions.

### Readmission

In a large US study of the Nationwide Readmissions Database (2013–2014) of 6058 DBS surgeries, the 30-day non-elective readmission rate was 4.9% [45], while interestingly, in another analysis of the same database (2016–2017), it was found to be 15.5% for PD patients and 7.8% for ET patients [49]. The former is slightly lower than our 6.9% (19/276) but our window extends to six months, as we found infections and side-effects of stimulation may not present in the first 30 days. This is especially relevant to our centre’s practice, where the IPG is activated four to six weeks after the implantation procedure. Also, in the latter database study, 11.5% of PD patients and 6.5% of ET patients required revision within 30 days, which is much higher than our experience [49]. Our study is consistent with another smaller study of 347 DBS procedures measuring 6.6% 30-day readmission, primarily due to infection, haematoma, seizures, altered mental status [39].

Importantly, in the current study, a six-month timeframe was used rather than 30 days, since more than 50% (15/27) were actually readmitted more than 30 days post-discharge. While our percentage may be slightly higher, it captured a more comprehensive set of data. Considering the cost of readmission using UK-based data, the cost of all readmissions (excluding planned ones) would be around £326 000 [15, 38], which is substantial in a publicly-funded system.

In addition, previous studies found lower socioeconomic status, higher comorbidity burden, greater frailty, and teaching hospital status to be associated with a higher rate of readmission [17, 21, 39, 45]. Yet, others found social deprivation was not necessarily a significant factor [19]. In the current study, social status was not an associated factor but the difference in demographics and how healthcare is reimbursed between countries may play a factor. With regards to teaching hospital status as a risk factor, given almost all UK hospitals with DBS surgical service are tertiary centres, teaching hospital status is universal in our setting. Further, it is possible non-teaching hospitals may select certain lower risk cases, rather than being a protective factor. While distance to hospital was not found to be a significant factor for readmission within six months in the current study, promising advancing technology such as remote programming and adaptive DBS system may help to streamline programming and reduce potential side-effects from the DBS system, and hence need for readmission [11, 13, 35, 47].

In the current study, the use of a skull-mounted device was associated with increased readmission. The recorded reasons included symptomatic haemorrhage (two cases), infection (one case), cerebral oedema (one case), and wound dehiscence (one case), with only the wound dehiscence case requiring reoperation. This may indicate potentially a learning curve and experimental nature in the implantation of these devices, which require further drilling of the skull to accommodate the IPG. It will be of interest to see if an increased complication rate is found in the other studies with this kind of devices [12]. Patient should be counselled on the possible increased risk of readmission in relevant clinical trials.

### Limitations

This is a retrospective study with a limited number of patients, which limits the validity of the conclusions. However, it spans over eight years and provides a balanced mix of patients. There are missing values in the hospital records, which may potentially introduce systemic bias, although this is likely to be random due to documentation and filing issues. While this is a single-centred study, the patient population and operating technique are likely more homogenous than those in large national databases mentioned in the Discussion.

In the current study, we have defined “increased” LoS as more than two nights. While this may be arbitrary, the literature mostly quotes average stay as one to three nights as mentioned in the Discussion. Together with our own experience, a cut-off of two nights appears pragmatic. Correlation analysis was also included in our Results to address that.

Furthermore, the outcome presented are the results of our centre’s technique and patient selection. DBS can be performed in a number of different ways, e.g. frame-based vs. frameless [40] and awake vs. asleep techniques [18]. Our centre also has formalised protocols in assessing and optimising patients before their operations [32]. These may be not generalisable to other units’ practice, which may have very different workflow and practice. For example, some centres perform DBS in two stages (insertion of electrodes and insertion of IPG) and some prefer to activate and programme the IPG in the index admission, potentially prolonging the in-patient stay. We also preferentially targeted the STN in PD cases, with only 2 cases involved the globus pallidus internus (GPi). This is not always in the case in other centres.

In addition, the UK has a predominantly publicly-funded healthcare system. Therefore, our results are not generalisable to other countries that have a strong private market, such as the USA.

## Conclusions

In the current study, we have identified the modifiable and non-modifiable factors associated with prolonged stay and readmission in patients who were treated in our DBS workflow. It was found old age and use of antiplatelets/anticoagulation were associated with prolonged length of stay in hospital after DBS insertion in PD patients. Use of skull-mounted device was associated with patient readmission within six months. The commonest cause was post-operative infection. These results would form the basis in enhancing patient selection and prehabilitation, as well as patient counselling and allocation of healthcare resources. Further studies with other workflow and healthcare systems would clarify the generalisability of our findings.

## Data Availability

Anonymised data may be available upon request.

## Abbreviations

AMES: Automated Memory and Executive Screening
ASA: American Society of Anesthesiologists
CTS: Clinical Tremor Score
DBS: Deep brain stimulation
DR-2: Dementia Rating Scale–2
ERAS: Enhanced recovery after surgery
ET: Essential tremor
IPG: Implantable pulse generator
LL: Long stayers
LoS: Length of stay
MMSE: Mini-Mental State Examination
NG: Nasogastric tube
NHS: National Health Service
PD: Parkinson’s disease
PDQ-39: Parkinson's Disease Questionnaire-39
SL: Short stayers
UPDRS3: Unified Parkinson's Disease Rating Scale-3
